# Immune response, oxidative stress, and histological changes of Wistar rats after being administered with *Parascaris equorum* antigen

**DOI:** 10.1038/s41598-024-67788-y

**Published:** 2024-08-05

**Authors:** Salma Adel Yehia, Abeer Mahmoud Badr, Abdel-Rahman Bashtar, Mahmoud Abdel-Aziz Ibrahim, Mohamed Refat Mousa, Nesma Abbas Mostafa

**Affiliations:** 1 Zoology Department, Faculty of Science, Cairo University, Giza, Egypt; 2 Pathology Department, Faculty of Veterinary Medicine, Cairo University, Giza, Egypt

**Keywords:** *P. equorum*, Wistar rats, Cytokines, CBC, Oxidative stress, Histopathology, Biochemistry, Biotechnology, Immunology, Zoology

## Abstract

Worldwide, particularly in developing nations, helminth infections are the leading causes of livestock illness and mortality. *Parascaris* (P.) *equorum,* a parasitic worm from the Ascarididae family, significantly impacts the production, health, and working performance of equines. This study aimed to investigate the impact of intraperitoneal sensitization of *P*. *equorum* on the immune system, oxidative stress, and histology in Wistar rats. After acclimatization for 7 days, we divided the rats into five groups, each consisting of six rats. Group I, serving as the control, was administered distilled water, followed by groups II (day 7), III (day 14), IV (day 21), and V (day 33). The rats were euthanized every day mentioned (Days 7–33). On day 0, a dosage of 1ml/100 gm rat (containing 500 μg/ml protein content) emulsified crude antigen extract with an incomplete Freund’s adjuvant (1:1 volume), followed by a second dose of the same antigen concentration on day 7. To assess the allergenicity of this nematode, we measured a whole blood profile, serum levels of IFN-γ, IL-5, IL-10, IL-13, and IL-33, total immunoglobulins IgE and IgG, and oxidative stress markers. Also, we examined histological changes in the liver, kidney, and spleen. The results showed that values of total leukocyte count, granulocytes, monocytes, and lymphocytes were significantly (*P* < 0.05) increased on day 14 post-infection relative to other days of investigation. It was found that the levels of total immunoglobulins (IgE and IgG) and cytokines (INF-γ, IL-5, IL-13, and IL-33) on days 14 and 21 were significantly higher than in the control group. At all periods of the experiment, the injected group exhibited significantly higher concentrations of MDA and NO compared to the control group (*P* < 0.05). Conversely, GSH and CAT levels (*P* < 0.05) dropped significantly on days 7, 14, and 21. Different rat tissues showed alterations. Ultimately, this study described the detrimental effects of *P. equorum* crude antigen administration on the immune system, oxidative states, and histological changes of Wistar rats at various intervals.

## Introduction

Infection with gastrointestinal nematodes is a significant health problem, impacting millions of humans and domestic animals, and can result in substantial economic losses in livestocks^1–4^. *Parascaris* (*P.*) *equorum* is considered the most pathogenic parasite, infecting the small intestines of horses and other equids via contaminated feed with infected eggs disseminated in the environment^5^. Mirian et al.^6^ reported that the infection prevalence of horses (*Equus caballus*) with this parasite ranged from 12.2 to 40.0%. Several clinical conditions develop upon infection, such as respiratory distress, nasal discharge, reduced appetite, weight loss, diarrhea, and colic^7^. Furthermore, the infected animals have dull hair and a rusty appearance^5^. Impaction of the small intestine is a significant concern associated with this parasite, frequently needing surgical intervention, and can cause death^7,8^. The extent of damage they can inflict varies according to number of parasites, nutritional status, and immune system of equids^3^. Understanding the relationship between hosts and their parasites, particularly the immune response, is necessary to develop sustainable nematode control strategies^9^. The Th2 response is responsible for resistance to gastrointestinal nematodes by producing the cytokines IL-4, IL-5, and IL-13, leading to elevated immunoglobulin IgE levels and the activation of specific effector cells, such as mast cells, eosinophils, and basophils, which is crucial for the immune eradication of nematodes and controlling infections^10^. During the initial stages of immune response to parasitic nematode infection, interferon (IFN)-γ and tumor necrosis factor (TNF)-α stimulate the activation of phagocytic cells, including granulocytes, monocytes, and macrophages, leading to the production and release of superoxide anion (O2 • −) and hydrogen peroxide (H_2_O_2_) to combat infection^11,12^. This activation may produce excessive reactive oxygen species (ROS), such as nitric oxide (NO), which is more toxic for the worm^13^. Therefore, it contributes to the immunologically mediated defense against many parasites^14^. Nevertheless, excessive and sustained generation of ROS can lead to oxidative stress, damage host tissues, and contribute to the overall pathology associated with the infection^15,16^. Moreover, reactive oxygen species (ROS) within the host of a parasitic infection can harm biomolecules such as lipids, proteins, and nucleic acids^16^, thereby destroying cells' structural and functional integrity. However, the enzymatic antioxidants may protect host cells from the many free radicals that result from parasitic infections^17^.

Based on the problems accompanied by this nematode infection in domestic horses as the main predominant nematode responsible for parasitic gastroenteritis, we designed this work to explore the impact of exposure to this nematode antigen extract in Wistar rats and give a whole picture of the level of immune response, oxidative stress status, and histological changes along different periods of experiment, that might be a guide to finding the effective anthelminthic agents.

## Materials and methods

### Worm collection and parasitological study

The alimentary tracts of domestic horses (*Equus ferus caballus* Linnaeus 1758; Family Equidae) were necropsied for educational purposes at the Faculty of Veterinary Medicine, Cairo University, Egypt. Each organ of the tract was isolated, placed in separate shallow plastic jars filled with normal saline (0.85%), and then transported to the Parasitology laboratory in the Zoology Department of the Faculty of Science. As described by Boomker et al.^18^, the contents of the intestine and stomach were transferred to distinct plastic containers filled with water. We thoroughly combined the contents and the mixture using a glass pipette. Different portions were placed in petri dishes and observed under a dissecting microscope. The retrieved worms were rinsed in physiological saline and 10% acetic acid multiple times to eliminate residual mucus or host debris. The recovered worms were washed in physiological saline and then in 10% acetic acid to remove any mucus or host debris. Five to ten millimeters of the cephalic and caudal extremities were cut off with a blade and rinsed in a lactophenol solution to prepare individual worms for light microscopy. Photomicrographic images were obtained by a LEICA DM 750 microscope equipped with a LEICA ICC 50 HD camera. Worms were subjected to SEM according to the following procedures: fixation in a 3% glutaraldehyde solution, subsequent washing in a 0.1M sodium cacodylate buffer (pH 7.4), dehydration via a graduated ethanol series (50%, 60%, 70%, 80%, 90%, and 100%), and drying at 30 °C for 30 min utilizing a critical point drier (LEICA, EM CPD300). Using an accelerating voltage of 25 kV, dried specimens were examined with a JEOL JSM-5200 SEM (Tokyo, Japan) after being mounted on stubs and coated with gold. Body dimensions were expressed as means (mm ± S.E). The parasite species were identified according to the keys of Lichtenfels^19^.

### Preparation of nematode crude antigen

Nematode worms (3–5) were homogenized in PBS solution by a pestle homogenizer. 10–20 µl of a protease inhibitor mixture was added to the worm homogenate. The suspension was centrifuged for 20 min at 10,000 rpm at 4 °C in a cooling centrifuge, and the supernatant was transferred to a sterile, clean tube while the pellet was removed. The crude extract's protein concentration was estimated by the Bradford^20^ method, and the extract was kept at − 20 °C in aliquots until used.

### Experimental design

Thirty male Wistar rats, *Rattus norvegicus*, aged 7–8 weeks old, weighing about 150–170 gm each, were bought from the National Organization for Drug Control and Research and subsequently relocated to the Laboratory of Parasitology, Zoology Department, Faculty of Science, Cairo University. Rats were kept at ambient temperature under a 12-h light–dark cycle and provided with ad libitum water. They fed a daily commercial diet consisting of 48% carbohydrates, 27% proteins, 7% fat, 8% fiber, and 9% minerals and vitamins^21^ purchased from the National Organization for Drug Control and Research.

After acclimatization for 7 days, the rats were divided into five groups, each consisting of six rats. Group I, serving as the control, was administered distilled water. According to Erhirhie et al.^22^, following OECD's (organization of Economic Corporation and Development) guidelines, the drug should not exceed 10 ml/kg (1 ml/100g) body weight of the experimental animals (rats and mice). Groups II to V on day 0 were experimentally injected intraperitoneally with 1 ml/100 gm rat (containing 500 μg/ml protein content) crude antigen extract emulsified in an incomplete Freund's adjuvant (1:1 volume). Afterward, the rats were boosted by the same dose with complete Freund’s adjuvant (1:1 volume) on day 7. The enhanced animals were euthanized by sodium pentobarbital (50mg/kg body weight^23^) on the following days: day 7 (group II), day 14 (group III), day 21 (group IV), and day 33 (group V).

### Blood Sample collection

Blood samples were taken in heparinized tubes to determine different hematologic parameters using an automated hematology analyzer (HA-Vet, Clindiag 202 System, BVBA, USA). Also, serum samples were prepared by centrifuging blood for 20 min at 3000 rpm, then kept at − 80 °C for immunological analyses.

### Cytokines and antibody levels

The levels of interleukins (IFN-γ, IL-5, IL-10, IL-13, and IL-33) and antibodies (total IgE and IgG) in serum were measured using an enzyme-linked immunosorbent assay (ELISA) kits by the manufacturer's instructions (SUNLONG Biotech Co., Inc., China). We measured all optical densities at 450 nm. The calculated overall intra-assay coefficient of variation was < 10% and the intra-assay coefficient of variation was < 12%. The concentrations of antibodies and cytokines were expressed in pg/ml.

### Assessment of oxidative stress markers

Hepatic rat tissues from all tested groups were isolated and homogenized (10% wt/vol) in an ice-cold 0.1 M Tris HCl buffer (pH 7.4), centrifuged for 10 min at 3000 rpm, and the resulting supernatant was kept at -20°C until used. The lipid peroxidation marker, malondialdehyde (MDA), was assessed as outlined by Buege and Aust^24^, and nitric oxide (NO) was determined according to Montgomery and Dymock^25^. The activity of the antioxidant enzyme catalase (CAT) was determined as described by Aebi^26^, and the concentration of glutathione reduced (GSH) estimated based on the method described by Beutler et al.^27^. Oxidative stress biomarker levels were calculated using reagent kits purchased from Bio-Diagnostic (Egypt).

### Histopathological investigation

Different rat tissues (Liver, kidney, and spleen) were isolated, cleaned with physiological saline, and fixed with 10% formalin for 24 h. Following that, as described by Nanji et al.^28^, the samples were dehydrated in an ascending series of alcohol concentrations, treated with xylene for clarity, embedded in paraffin, and then sectioned to a thickness of 5 µm. Hematoxylin and eosin (H&E) were used to stain the sections before histologic examination under a light microscope to detect histological changes such as congestion, hemorrhage, cell necrosis, edema, and hepatocyte vacuolization.

### Statistical analysis

Data were expressed as means ± standard error of the mean (SEM) for six animals in each group. One-way analysis of variance (ANOVA) was employed to assess the differences between groups, followed by a Duncan post hoc test using SPSS version 20 (SPSS Inc., Chicago, IL, USA) software. *P* values less than 0.05 were regarded as statistically significant.

### Ethical approval and consent to participate

The current study was conducted in accordance with the relevant guidelines and regulations of ARRIVE guidelines. The Cairo University Institutional Animal Care and Use Committee (CU-IACUC) approved all experimental procedures using animals in this study under the relevant document (No. CU/I/S/44/16). All laboratory animal use procedures in this study were agreed upon according to the Ethics of Research Committee regulations at the Faculty of Science, Cairo University, and received the approval number (No. CU/I/S/44/16).

## Results

### Morphological description of the nematode worms (based on 5 specimens, Figs. 1 and 2)

**Figure 1:**
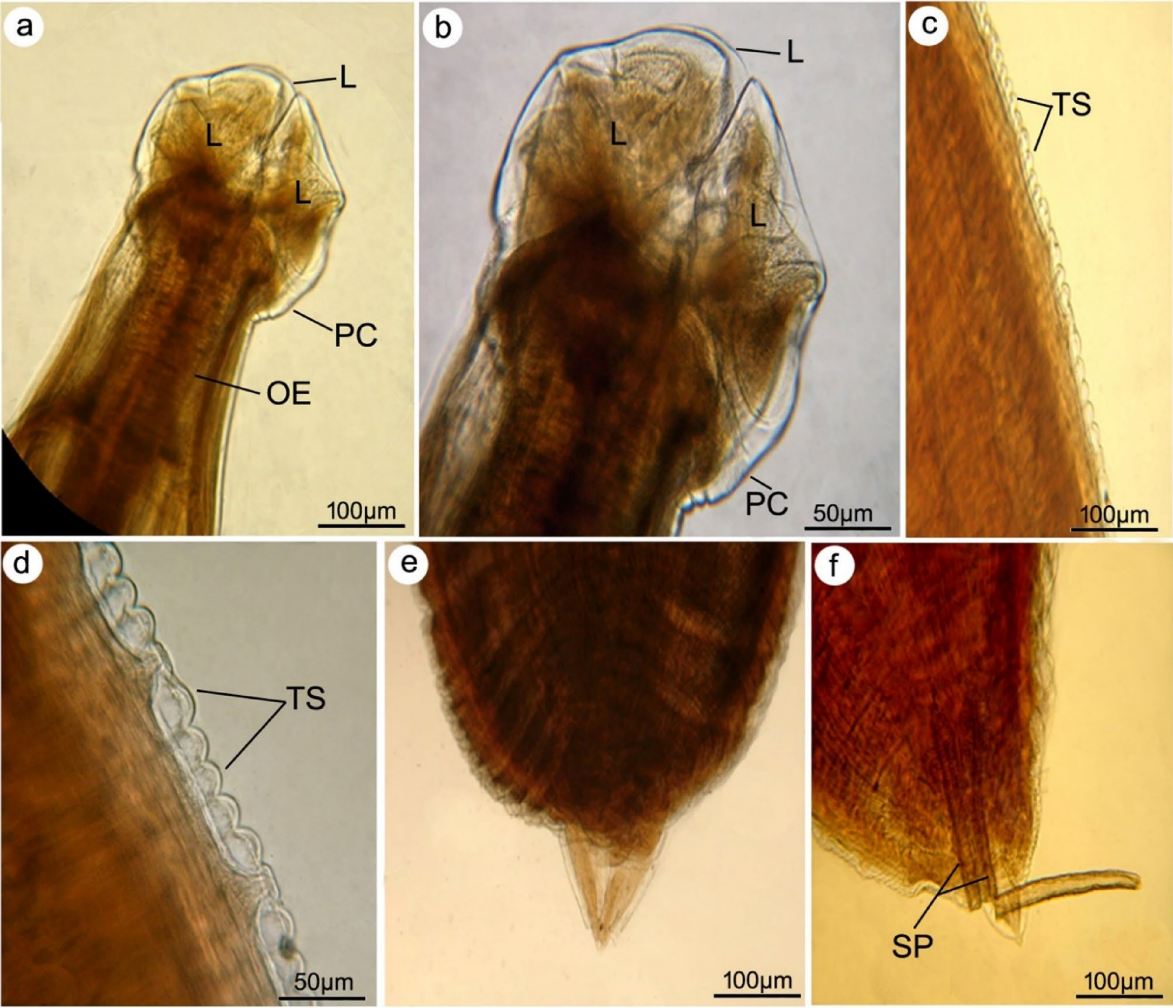
Photomicrograph of adult nematode, *P. equorum* cleared with lactophenol. (**a**,** b**) Anterior extremity of worm showing 3 lips (L) one dorsal, and two subventrals forming interlabia (IL) in between and separated from the body by a deep post-labial constriction (PC), mouth opening (MO), and muscular esophagus (OE). (**c**,** d**) Transverse striations (TS) of the body cuticle sharply defined. (**e**) The female has a conical tip. (**f**) Male posterior ends with two short spicules (SP).

**Figure 2:**
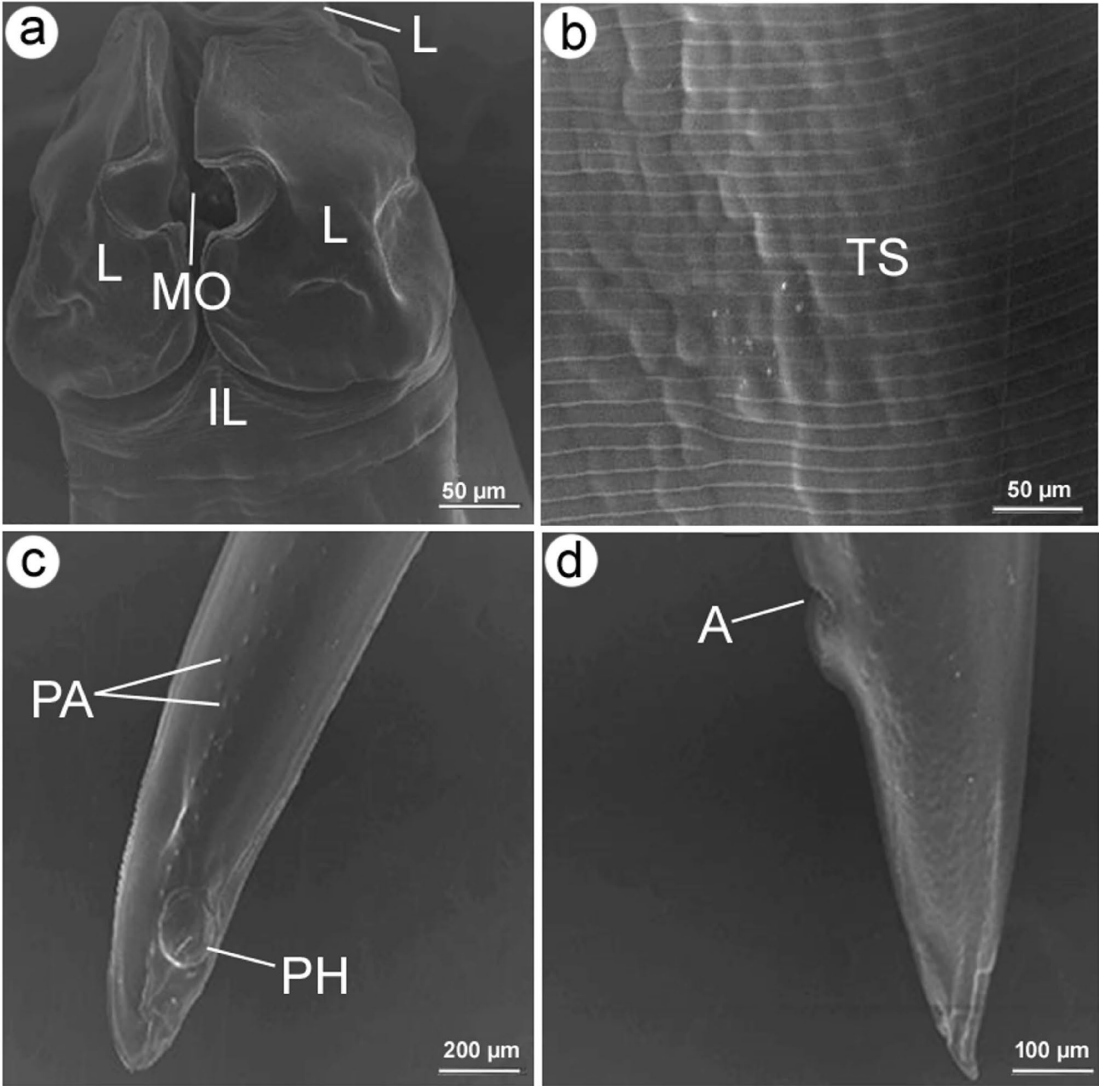
Scanning electron micrographs of *P. equorum*. (**a**) The adult worm's anterior end displayed three interlocked lips (L), which were organized in the form of one dorsal and two sub-ventrals around the mouth opening (MO) and creating an inter-labium (IL) in between. (**b**) Transverse striations of the cuticle (TS). (**c**) Button-like phasmid (PH). (**d**) Conical end of a female with a prominent anus (A).

At the macroscopic level, the worms retrieved from the gastrointestinal tract of the dissected horse specimen exhibited elongated, white, and unsegmented characteristics. With the naked eye, male and female worms were distinguishable by their wrapped and upright ends. Males measured 15 ± 2 (12–17) cm in length, while females measured 17 ± 2 (14–20) cm. Light and scanning electron micrographs revealed that both sexes have broad anterior ends with three interlocked lips that resemble a shamrock, two sub-ventral and one dorsal, and two or more cephalic papillae surrounding the mouth opening. Deep post-labial constriction separated the labia from the rest of the body, leaving a triangular-shaped inter-labia in between. The cuticle was transversely striated and contained prominent, narrow annules. There were no caudal alae seen. The male tail was long and characterized by a triangular shape and a pointy tip, adorned with many papillae organized in longitudinal rows supplied with two spicules. At the same time, the female had a conical tail that was somewhat attenuated at the distal third.

### The effects of worm administration

#### Hematologic findings

As demonstrated in Table 1, values of total leukocyte count and differential cells, granulocytes, monocytes, and lymphocytes were considerably (*P* < 0.05) raised on day 14 post-injections relative to other days. Hemoglobin content declined on day 14, and red cell count and hematocrit increased significantly on days 7, 21, and 33. Additionally, MCV revealed a significant increase on day 7. While MCH revealed no significant change throughout the entire day of investigation, in terms of platelet count, it was relatively lower on day 7 compared to the control but significantly higher on days 14, 21, and 33. MPV significantly increased on days 7, 14, and 21 while returning to normal on day 33. Throughout the entire days of investigation, PDW showed no significant change. PLCC significantly increased on days 7, 14, and 21 while returning to normal on day 33.

**Table 1:** Complete blood analysis of rats intraperitoneally injected with crude antigen extract of *P. equorum* at different time points compared to control.

Hematologic parameters	Control group	Injected rat groups			
		Day7	Day14	Day 21	Day 33
WBCs (10^3^/cm)	12.43 ± 0.56^a^	9.733 ± 2.47^a^	139.3 ± 14.49^b^	15.40 ± 2.15^a^	18.83 ± 0.77^a^
Gran (10^3^/cm)	6.44 ± 1.89^a^	4.71 ± 1.79^a^	28.67 ± 1.15^b^	1.484 ± 0.431^a^	0.948 ± 0.37^a^
Mon (10^3^/cm)	0.293 ± 0.055^a^	0.21 ± 0.10^a^	9.67 ± 0.47^b^	0.94 ± 0.45^a^	1.86 ± 0.54^a^
Lym (10^3^/cm)	3.36 ± 0.43^a^	4.34 ± 0.66^a^	65.92 ± 3.09^b^	6.65 ± 3.55^a^	12.33 ± 3.24^a^
HB (gm/dl)	13 ± 0.28^a^	13.96 ± 0.54^a^	11.26 ± 0.31^b^	12.8 ± 0.28^a^	12.96 ± 0.28^a^
RBCs (10^3^/cm)	5.93 ± 0.68^a^	7.58 ± 0.39^b^	5.86 ± 0.26^a^	6.57 ± 0.17^a b^	6.81 ± 0.07^a,b^
Hematocrit (PCV) %	32.00. ± 0.57^a^	37 ± 1.52^b^	34.46 ± 0.40^a,b^	36.53 ± 0.55^b^	36.2 ± 0.72^b^
MCV (fL)	55 ± 2.51^a^	66 ± 3.05^b^	54.66 ± 1.20^a^	56 ± 2.30^a^	56.66 ± 3.71^a^
MCH (pg)	20.33 ± 0.66^a^	20.66 ± 1.16^a^	19 ± 0.57^a^	19.66 ± 0.88^a^	20.33 ± 1.33^a^
Platelet (10^3^/cm)	251.33 ± 10.47^a^	193.33 ± 32.64^a^	930.66 ± 255.42^b^	729.66 ± 99.46^b^	578 ± 42.53^a,b^
MPV (f L)	6.1 ± 0.05^a^	7.1 ± 0.152^b^	6.76 ± 0.266^b,c^	6.56 ± 0.145^a,b,c^	6.13 ± 0.066^a,b^
PDW %	15.1 ± 0.10^a^	16.43 ± 0.39^b^	15.30 ± 0.20^a^	15.13 ± 0.120^a^	15.06 ± 0.088^a^
PLCC (10^9^/L)	24.33 ± 2.72^a^	35.00 ± 1.73^a,b^	47.33 ± 9.33^b^	42.33 ± 2.02^b^	23.33 ± 2.84^a^

Data is presented as mean ± SEM (n = 6), where values with different superscript letters are regarded as statistically significant (*P* < 0.05). WBCs, white blood cells; Gran, Granulocytes; Mon, Monocytes; Lym, Lymphocytes; Hb, hemoglobin; RBCs, Red blood cells; PCV, packed cell volume; MCV, mean cell volume; MCH, mean cell hemoglobin; MPV, mean platelet volume; PDW, red cell distribution width; PLCC, Platelet-large cell count.

### Immunological profile

The current study used the ELISA technique to identify the humoral immune responses against the experimental infection of Wistar rats with *P. equorum* crude antigen extract. Table 2 indicated that the levels of IFN-γ, IL-5, and IL-13 were significantly higher (*P* < 0.05) in the injected groups on days 14 and 21 compared to the control rats. However, these levels returned to normal on day 33 following the injection. IL-33 was significantly (*P* < 0.05) elevated on days 7, 14, and 21. Meanwhile, IL-10 showed no significant change among all the examined groups. The concentrations of total IgE were significantly higher (*P* < 0.05) on days 14, 21, and 33 compared to the control group. Similarly, the levels of total IgG considerably increased (*P* < 0.05) on days 14 and 21 and recovered to average values on day 33 post-injection.

**Table 2:** Levels (pg/ml) of serum cytokines (INF-γ, IL-5, IL-10, IL-13, IL-33) and antibodies (IgE, IgG) in the control group and rats intraperitoneally injected with crude antigen extract of *P. equorum* at different time points.

Immunologic parameters	Control group	Injected rat groups			
		Day 7	Day 14	Day 21	Day 33
INF-γ	275.74 ± 19.97^a^	374.18 ± 14.09^a^	864.42 ± 216.47^b^	919.28 ± 197.96^b^	417.04 ± 68.25^a^
IL-5	237.76 ± 15.35^a^	293.316 ± 31.25^a^	1078.21 ± 197.23^b^	1036.07 ± 206.35^b^	286.68 ± 18.17^a^
IL-10	12.98 ± 1.61^a^	12.77 ± 1.33^a^	9.94 ± 1.79^a^	14.16 ± 1.72^a^	11.43 ± 1.79^a^
IL-13	565.41 ± 69.46^a^	601.42 ± 59.95^a^	1686.66 ± 321.95^b^	1763.33 ± 231.72^b^	918.09 ± 95.99^a^
IL-33	292.22 ± 23.19^a^	479.52 ± 16.87^b^	447.77 ± 50.67^b^	472.22 ± 52.57^b^	308.88 ± 60.69^a^
Total IgE	922.5 ± 163.32^a^	1077.85 ± 131.91^a,b^	1430 ± 158.58^b^	1470 ± 110^b^	1360 ± 92.42^b^
Total IgG	44.39 ± 27.89^a^	63.39 ± 14.06^a^	164.39 ± 18.82^b^	145.30 ± 16.70^b^	74.22 ± 12.39^a^

Data is presented as mean ± SEM (n = 6), where values with different superscript letters are regarded as statistically significant (*P* < 0.05).

### Oxidative stress biomarkers

**Figure 3:**
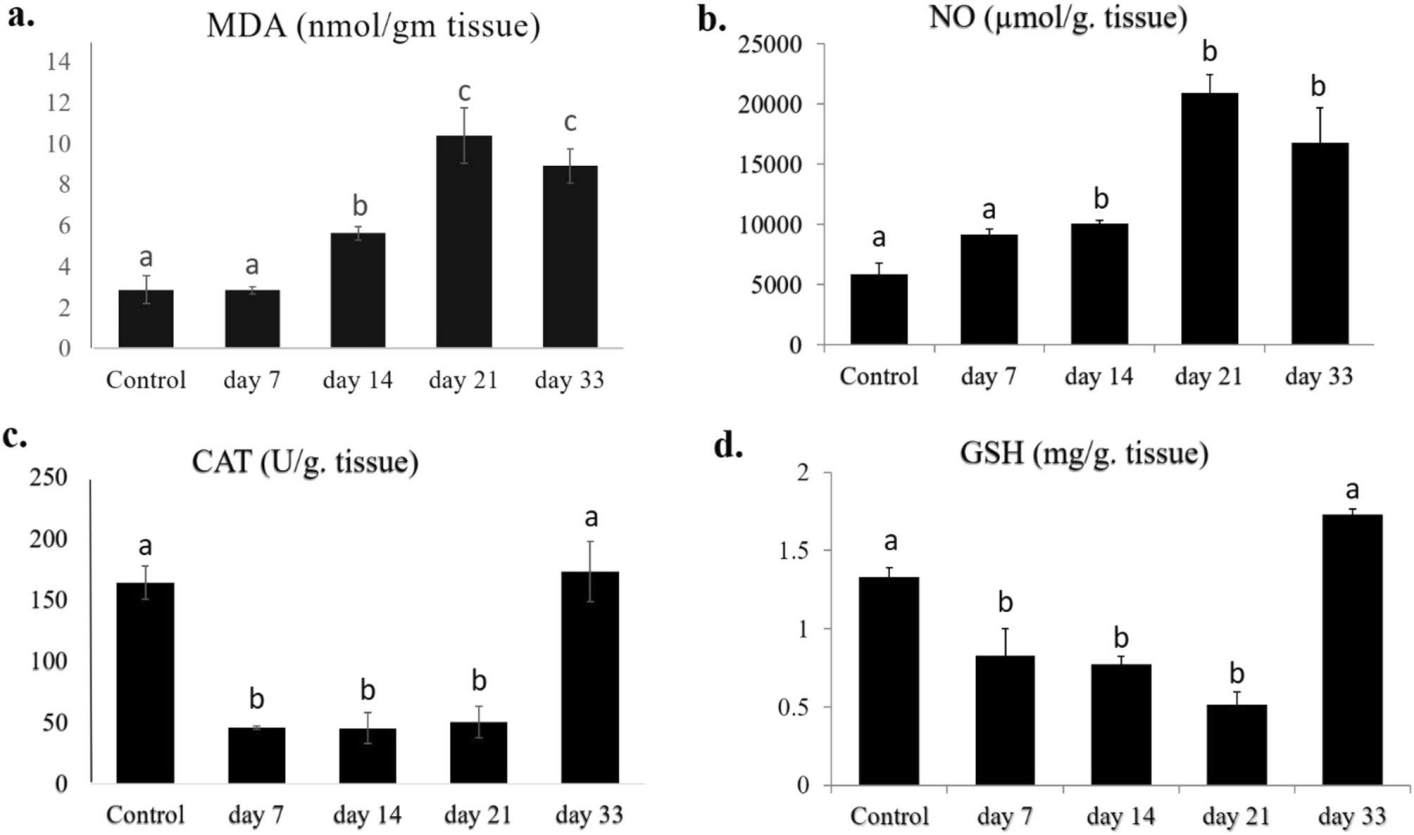
Hepatic levels of CAT, GSH, MDA, and NO in rat groups injected with *P. equorum* crude antigen on days 7, 14, 21 and 33 post-injections compared to control group. Data is presented as mean ± SEM (n = 6), where values with different superscript letters are regarded as statistically significant (*P* < 0.05).

Figure 3 revealed that the intraperitoneal sensitization of Wistar rats by *P. equorum* crude antigen led to oxidative stress in the hepatic tissue, as evidenced by a notable increase in MDA and NO levels in the injected group compared to the control group (*P* < 0.05) through all different time intervals post-infection. Also, a substantial (*P* < 0.05) decrease in GSH and CAT concentration was detected on days 7, 14, and 21 post-injections, returning to a normal state at day 33, like the control.

### Histopathological findings

**Figure 4:**
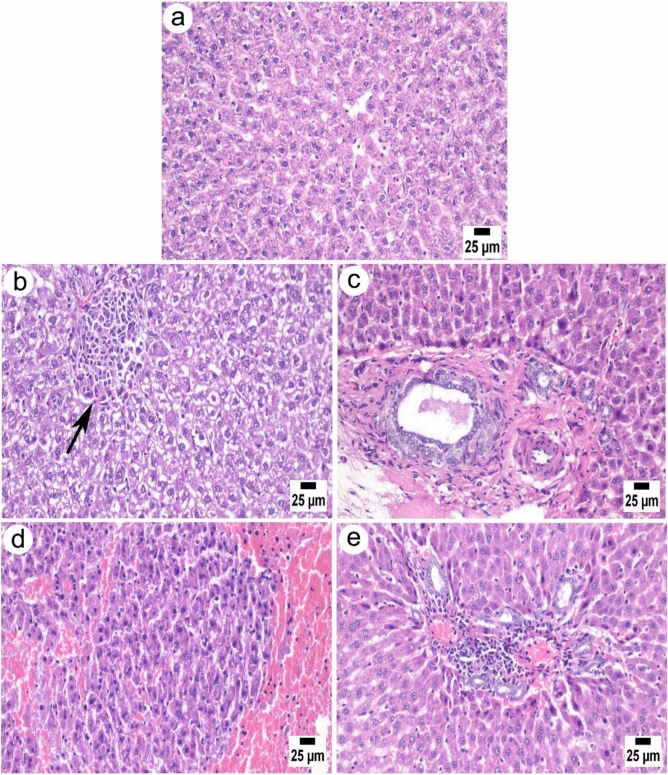
Photomicrographs of liver sections of Wistar rats. (**a**) The control group showed normal healthy polygonal hepatocytes (H) with rounded vesicular nuclei and acidophilic cytoplasm. (**b–e**) Intraperitoneal injected rats investigated at different time points, (**b**) Group II (day 7) showing vacuolated hepatocytes with aggregation of inflammatory cells replaced necrotic hepatocytes (arrow). (**c**) Group III (day 14) showing portal fibrosis with ductular epithelial hyperplasia. (**d**) Group IV (day 21) showing severe hepatic hemorrhage. (**e**) Group V (day 33), focal portal hepatitis was detected. All sections were stained with H&E, magnification 400x, and a scale bar of 25µm.

Microscopic examination of the liver of control rats appeared normal, with orderly arranged hepatocytes in normal lobular architecture (Fig. 4a). In contrast, on day 7, liver sections of injected rats showed a multifocal area of coagulative necrosis of the hepatic parenchyma associated with the inflammatory cell aggregations. (Fig. 4b). On day 14, the liver tissue showed marked portal fibroplasia associated with bile ductular epithelial hyperplasia (Fig. 4c). The multifocal areas of mononuclear inflammatory cell aggregations in the hepatic parenchyma and hemorrhages were frequently detected on day 21 (Fig. 4d). Finally, on day 33 post-injection, the microscopic examination of the liver sections also showed multifocal hepatitis areas characterized by the accumulation of mononuclear inflammatory cells in the hepatic lobules and the portal areas, accompanied by severe hemorrhagic areas with excessive erythrocyte exudation replacing the hepatic parenchyma (Fig. 4e).

**Figure 5:**
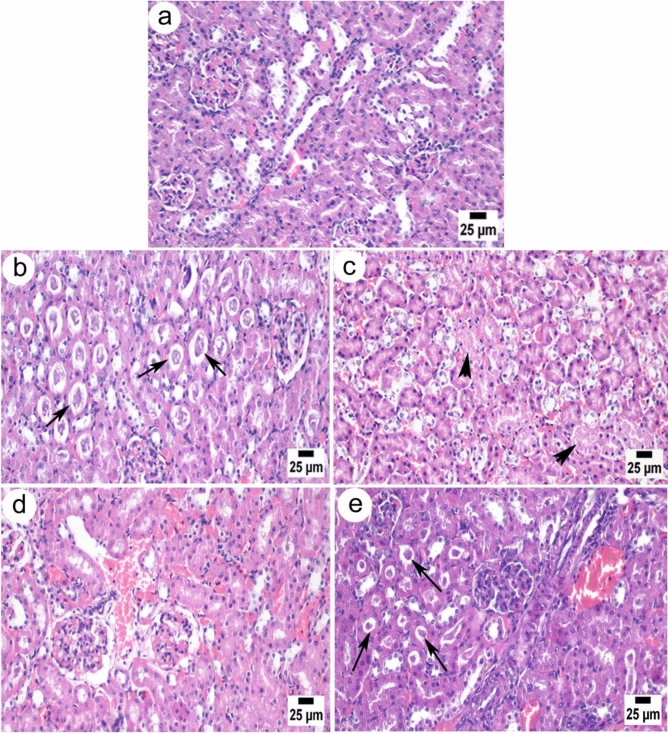
Photomicrograph of kidney sections of Wistar rats. (**a**) The control group showed normal renal tubules in the cortex. (**b**) On day 7, injected rats showed casts in the renal tubules (arrows). (**c**) On day 14, injected rats showed severe necrosis of renal tubular epithelium (head arrows). (**d**) On day 21, injected rats showed congestion of the renal cortex. (**e**) On day 33, injected rats showed renal casts (arrows). All sections were stained with H&E, magnification 400x, and a scale bar of 25 µm.

Compared to the kidney tissue of control rats, which displayed a normal structure of both the renal cortex and medulla (Fig. 5a), kidney sections on day 7 following the injection showed fewer necrobiotic changes in the epithelial lining cortical renal tubules, with numerous casts in the lumen of several renal tubules (Fig. 5b). On day 14, it was observed that the renal tubules suffered from coagulative necrosis (Fig. 5c). Necrobiotic changes were still observed in the renal tubules with congestion on day 21 (Fig. 5d). On day 33, necrosis of renal tubules, congestion, and renal casts still observed (Fig. 5e).

**Figure 6:**
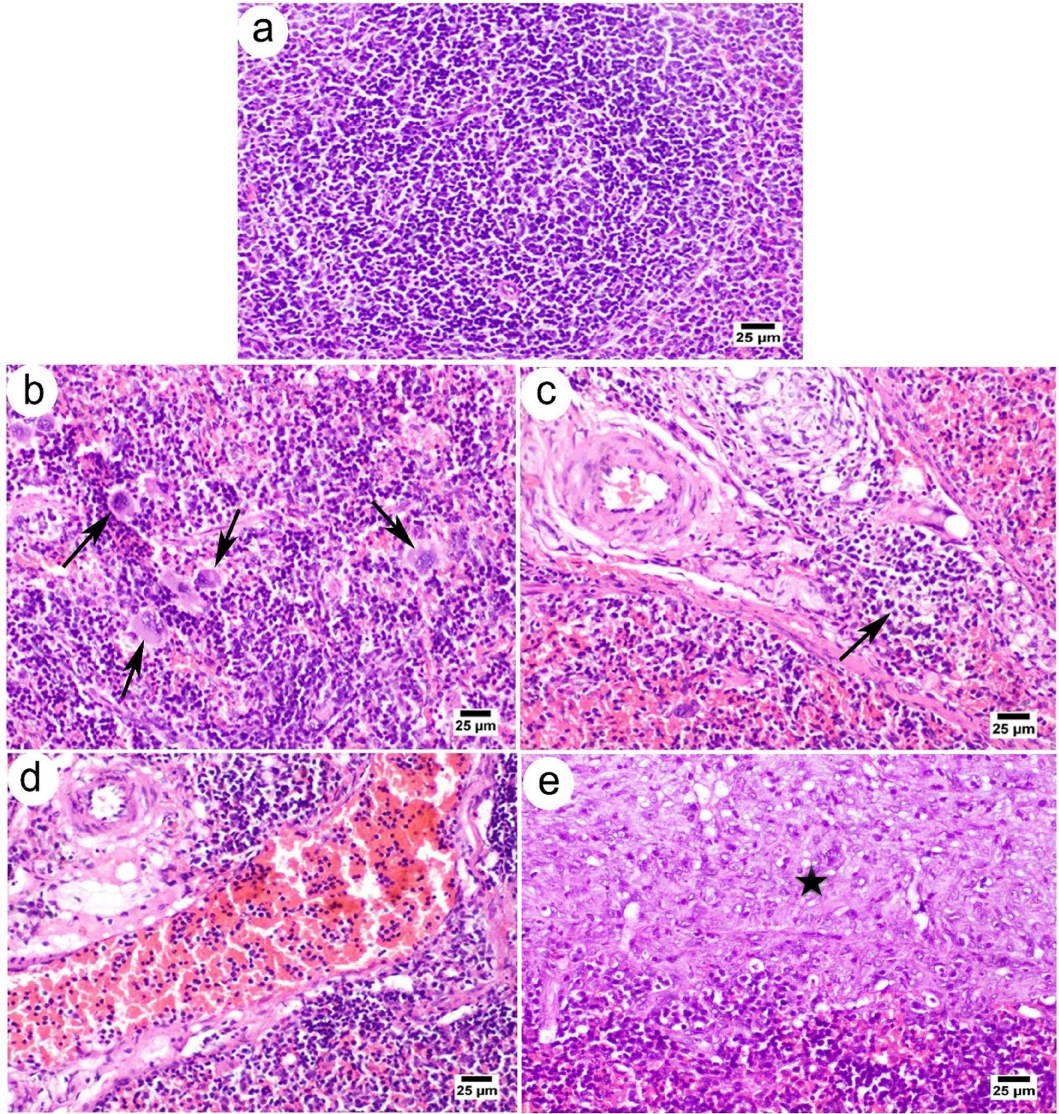
Photomicrographs of the splenic sections of Wistar rats. (**a**) Control group showing normal lymphoid tissue (white pulp) with germinal center (arrow). (**b**) On day 7, the injected group showed a variable number of megakaryocytes (arrows). (**c**) On day 14, injected rats showed inflammatory cell infiltration (arrow). (**d**) On day 21, injected rats showed congested blood vessels. (**e**) On day 33, injected rats showed severe thickening of the splenic capsules (asterisks) with excessive fibrosis and inflammatory cell infiltration. All sections stained with H&E, magnification 400x, and scale bar: 25 µm.

The splenic tissue of control rats exhibited a normal histology of splenic parenchyma formed of white and red pulps; splenic cords and sinusoids represented the red pulp, while the white pulp is composed of three compartments: the periarterial lymphatic sheath (PALS), lymphatic follicles, and the border zone (Fig. 6a). On the other hand, at day 7, splenic tissues showed some histopathological alterations, including the expansion of red pulps accompanied by mild atrophy of splenic follicles and existence of a variable number of megakaryocytes in some sections (Fig. 6b). Also, on day 14, perivascular edema was frequently detected in splenic tissue, accompanied by an increased number of inflammatory cell infiltration (Fig. 6c). On day 21, in addition to the expanded red pulps affected splenic white pulps, numerous congested blood vessels were noticed (Fig. 6d). On day 33, the splenic capsule showed severe thickening with abundant fibroplasia and intense mononuclear inflammatory cell infiltration (Fig. 6e).

## Discussion

Gastrointestinal parasitism has been identified as one of the most significant threats to equids in developing countries^29^. *Parascaris equorum* is a common ascarid nematode that inhabits the small intestines of equines, mainly young animals of horses and donkeys, which are highly susceptible for infection^30^. Using light and scanning electron microscopy, the morphological identification of the worm recovered in the current work indicated that it belongs to *P. equorum* by having a distinctive head region characterized by a shamrock-like three interlocked lips. This is consistent with the findings of studies published by Lichtenfels^19^. Additionally, Pilitt et al.^31^ noted that the labial and cuticular morphologies, the size and shape of the lip, and the spacing of the denticles surrounding the lip margins are the most valuable characteristics for differentiating morphologically similar developmental stages. Similar to the descriptions given by Morsy et al.^32^, it was observed that the cuticle of the current *P. equorum* is finely striated, containing a narrow annulus devoid of markings. Furthermore, the male possesses a shorter tail than the female, distinguished by two spicules of equivalent length. Phasmids were observed at the caudal end of females. Several studies have documented this severe parasite's morphological and morphometric description affecting foals and weanlings worldwide^7,33,34^.

Immunity during gastrointestinal parasite infections involves both humoral and cellular responses^35^, which are known to be closely dependent^36^. Although gastrointestinal nematode infections are globally prevalent, there is limited understanding of the specific mechanisms that generate protection against them^37^. The mechanisms underlying resistance to infection vary significantly between hosts and parasites^38^. However, the polarized type 2 response (IL-4, IL-5, IL-9, and IL-13) might reduce the severity of infection and contribute to the nematode parasite's eradication^10^. IFN‐γ is mainly secreted by activated T cells and natural killer (NK) cells and is considered the chief modulator of innate and adaptive immunity^39^. The current work demonstrated that crude antigen *P. equorum* promoted a significant rise (*P* < 0.05) of IFN-**γ** levels on days 14 and 21 post-injection while returning to normal values on day 33 compared to control rats. This finding is corroborated by Shlash et al.^40^, which reported higher levels of IFN-γ in *A. duodenale* infected patients.

IL-5 is produced by Th2 cells to stimulate antibody production from activated B cells after stimulation with allergens^41^. On days 14 and 21 following injection, it was observed that IL-5 significantly (*P* < 0.05) increased. In contrast, Tran et al.^42^ reported that *Nippostrongylus brasiliensis* infection induces eosinophilia and IL-5 production after 7 days, and parasite expulsion occurs after a rapid decline of IL-5 after 14 days. IL-13 possesses significant anti-inflammatory characteristics and is regarded as a pivotal cytokine in regulating pathogens, including helminthic parasites and allergic and inflammatory disorders^43^. Our data revealed that *P. equorum* crude antigen caused a significant increase in IL-13 levels on days 14 and 21 post-injection relative to other groups.

IL-33 is an anti-inflammatory cytokine and a potent stimulant of the innate immune system and plays a role in various diseases^44,45^. It is released when intestinal nematode larvae infect epithelium and cause damage as they move through the lungs and into the airways^46^. The level of IL-33 was linked to the number of worms^47^; lower levels of IL-33 in immunized mice were related to fewer lung larvae, less inflammation, and better function. Also, Humphreys et al.^48^ reported that IL-33 significantly contributes to eliminating bacterial and parasitic diseases. In addition, Pastorelli et al.^49^ reported that animal models of ulcerative colitis show elevated levels of IL-33. Thus, the current IL-33 high level of the injected groups on days 7, 14, and 21 suggested that it is a sign of protection against *P*. *equorum* crude antigen infection. In a similar vein, Neill et al.^50^ reported that nematode infection increased IL-33 levels. The cytokine IL-10 has crucial roles in modulating immune and inflammatory processes, which regulate inflammation by preventing the production of monocytes, macrophages, and numerous other proinflammatory cytokines and chemokines that stop inflammation-related tissue damage^51^. In the present study, IL-10 did not exhibit a significant difference among the groups, which explains the prominently observed inflammation in all different tissues.

The utilization of IgE and IgG immunoglobulins is critical in diagnosing various pathological states linked to bacterial, viral, and parasitic infections, allowing for the implementation of the correct treatment strategies^52^. There was a notable increase (*P* < 0.05) in the overall IgE and IgG concentrations of infected rats on days 14 and 21 compared to the control group. These results are consistent with Wright and Bickle^53^, who reported a rise in hookworm-specific IgG and IgE levels in humans following infection. The production of IgE is predominantly mediated by interleukins IL-4, IL-10, and IL-13 via the induction of B-cell switching^54^. Hence, the substantial increase in IgE levels in infected rats may be linked to elevated IL-13 levels rather than the examined IL-10, which did not exhibit any significant variation among the groups. As suggested by Cetre et al.^55^ and Mostafa et al.^56^, the elevated concentration of total IgG observed might be linked to the generation of INF- γ.

Parasitic infection can trigger oxidative stress, resulting in elevated ROS generation and reduced antioxidant levels^57^, which can damage membranes, DNA, and protein structures^58^. Overproduction of ROS leads to elevated levels of malondialdehyde (MDA), the primary product of lipid peroxidation^59^. It is considered the primary process by which ROS causes tissue damage and serves as a biomarker for oxidative stress^60^. Furthermore, inflammatory responses are accompanied by ROS production, which can impair vital functions and cause cellular death^61^. Compared to the control group, the current study revealed a substantial increase in MDA and NO concentrations on days 14, 21, and 33 following *P. equorum* crude antigen injection in rats, confirming that these nematode species induce oxidative stress and inflammation in accordance with a study conducted by Attia et al.^29^. CAT is the essential enzyme in reducing oxidative damage caused by free radicals; it contributes to the reduction of hydrogen peroxide to oxygen and water^62,63^. Intestinal parasitic infection inhibits the antioxidant system by lowering GSH levels. This drop was linked to the higher cytotoxicity of the generated H_2_O_2_ caused by the inhibition of glutathione reductase, which keeps glutathione in a reduced state^64^. Additionally, ROS generated by the parasites depletes the host's catalase (CAT) and glutathione (GSH)^65^. In this study, *P*. *equorum* antigen extract exerted a substantial (*P* < 0.05) suppressive impact on the level of CAT and GSH on days 7, 14, and 21 post-injection. These findings agreed with Derda et al.^66^, who noted that *T. spiralis* resulted in the breakdown of antioxidant enzyme systems in infected mice, disrupting metabolite balance through increased lipid peroxidation. Da Silva et al.^67^ also reported that *T*. *evansi* infection in camels reduced GSH content. Thus, it seems that the effect of intraperitoneal administration of this nematode antigen reflects an increase in oxidative stress related to intestinal nematode infection^68^.

Histopathological investigations displayed that *P. equorum* crude antigen extract caused severe changes in the infected rat tissues, including the liver, kidneys, and spleen, demonstrating a similar pattern of changes with time over days of infection. Liver sections showed inflammatory cell aggregations and portal fibroplasia associated with bile ductular epithelial hyperplasia. Moreover, hemorrhages were frequently detected replacing the hepatic lobules, and mononuclear inflammatory cell aggregation in the hepatic lobules and the portal areas, accompanied by severe hemorrhagic areas, was observed. These findings are consistent with Minemura et al.^69^ who report that the liver, when subjected to various infections caused by hepatotropic and non-hepatotropic pathogens, can enter the liver through the systemic and portal circulation. These pathogens can initiate liver damage, leading to diverse manifestations. Sections of the kidney showed less necrotic changes in the epithelial lining of the cortical renal tubules and more casts in the lumen of many of the renal tubules. It was observed that renal tubules suffered from coagulative necrosis. Necrobiotic changes were also observed in the affected renal tubules with congestion. Barsoum^70^ demonstrated that parasitic infections can cause kidney impairment.

The infected rats’ splenic tissue showed some histopathological alterations whereas the red pulps became more prominent, the splenic follicles became smaller, and there were different numbers of megakaryocytes. Perivascular edema was frequently detected, accompanied by an increased number of inflammatory cell infiltrations. Congested blood vessels were noticed, along with mononuclear inflammatory cell infiltration. The splenic capsule showed severe thickening, abundant fibroplasia and intense mononuclear inflammatory cell infiltration. Adhesion between the splenic parenchyma and adjacent organs was detected in several cases. These findings align with those of Fahmy and Diab^71^ who observed abnormalities in splenic lymphoid tissue associated with nematode infestation. Moreover, Fahmy and Diab^71^ and Ahmed et al.^72^ documented that tissue damage and inflammation were caused by elevated levels of reactive oxygen species (ROS) in infected rats. All the above-mentioned pathological alterations in rat tissues may be related to oxidative stress enhancement in histopathological sections.

## Conclusions

From the current study, it was concluded that sensitizing Wistar rats with *P*. *equorum* crude antigen extract led to inflammatory immune responses via increasing total leukocyte count on day 14 post-infection compared to other days of investigation and stimulating the production of antibodies, IgE and IgG, Th1- related cytokine (IFN-γ), and Th2 cytokines (IL-5, and IL-13), and innate immune responses (IL-33) particularly at days 7 and 14 post-injection. Furthermore, antioxidant markers, CAT, and GSH were downregulated, and elevated MDA and NO levels were observed on days 7, 14, and 21 following injection, indicating an oxidative stress state. Consequently, these conditions resulted in severe alterations in various histological sections, including the liver, kidney, and spleen, during the time period of the study. These findings provide a valuable screening perspective on the immune response to *P*. *equorum* infection. Nevertheless, further experimental studies are needed to recognize the specific antigens of this nematode that may be responsible for these adverse effects.

## Data Availability

Data are available upon request from the corresponding author.

## Abbreviations

A: Anus
L: Lips
IL: Interlabia
PC: Post-labial constriction
MO: Mouth opening
OE: Esophagus
TS: Transverse striations of cuticle
PA: Papillae
PH: Phasmid
CAT: Catalase enzyme
GSH: Reduced glutathione
MDA: Malondialdehyde
NO: Nitric Oxide
WBCs: White blood cells
Gran: Granulocytes
Mon: Monocytes
Lym: Lymphocytes
Hb: Hemoglobin
RBCs: Red blood cells
PCV: Packed cell volume
MCV: Mean cell volume
MCH: Mean cell hemoglobin
MPV: Mean platelet volume
PDW: Red cell distribution width
PLCC: Platelet-large cell count
